# SUMOylation balance: a key determinant in synapse physiology

**DOI:** 10.3389/fphys.2025.1675598

**Published:** 2025-10-01

**Authors:** Alessia Bertozzi, Walter Toscanelli, Giuditta Castellitto, Claudio Grassi, Claudia Colussi

**Affiliations:** ^1^ Department of Neuroscience, Università Cattolica del Sacro Cuore, Rome, Italy; ^2^ Istituto di Analisi dei Sistemi ed Informatica “Antonio Ruberti”, National Research Council, Rome, Italy; ^3^ Fondazione Policlinico Universitario Agostino Gemelli IRCCS, Rome, Italy

**Keywords:** SUMOylation, synapse, Alzheimer’s disease, post-translational modifications, neuron

## Abstract

Neuronal communication relies on the precise regulation of synaptic compartments, where protein activity, localization, and turnover are tightly controlled. Among the mechanisms ensuring this regulation, post-translational modifications (PTMs) play a central role. SUMOylation, the covalent attachment of Small Ubiquitin-like Modifier (SUMO) proteins to target substrates, has emerged as a dynamic key PTM in the nervous system, modulating synaptic structure and function. Target SUMOylation occurs through an enzymatic cascade and requires the presence of a consensus sequence. Reversible addition of SUMO monomers or chains may contribute to distinct functional outcomes changing the conformation of the protein thus favoring/inhibiting molecular interaction among proteins or stabilizing the protein inhibiting degradation or influencing subcellular localization. All these SUMO dependent effects are crucial in the regulation of the tiny and highly specialized synaptic compartments to achieve spatiotemporal control for proper neurotransmission and synaptic plasticity in response to environmental stimuli. Dysregulation of this system has been implicated in various neurological disorders, including Alzheimer’s disease, where imbalances in SUMO1 versus SUMO2/3 levels contribute to synaptic dysfunction. As such, comprehension of SUMO related mechanisms may give important insights into both physiological regulation of synapses and potential therapeutic approaches for neurodegenerative diseases. Thus, in this review we will first introduce the enzymatic cascade of SUMOylation and its impact on protein function, then we will focus on its role within the synaptic compartment. Finally, we will discuss the therapeutic potential of modulating SUMOylation in Alzheimer’s disease as example of neurodegenerative disorders.

## Introduction

The ability of neurons to transmit electrical impulse is based on their specialized structure that derives from a highly cellular polarization associated with the asymmetric distribution of proteins, RNA and organelles. Specifically, at the level of synapses, the functional units where an electrical signal is converted into biochemical changes, fine tuning of processes is achieved by multiple mechanisms based on proper temporal and spatial activation of the key players. Indeed, synapses are tiny but very complex compartments that modulate neurotransmission by altering their shape, size, and protein/RNA content. At the level of pre-synaptic zone, neurotransmitter release is based on the formation of different synaptic vesicle (SVs) pools grouped on the basis of their availability for exocytosis: (i) the readily releasable pool (RRP), comprising about 1% of SVs, which rapidly fuses with the plasma membrane in response to an action potential; (ii) a recycling pool, accounting for 10%–20% of SVs, which participates in neurotransmission during moderate physiological stimulation and replenishes the RRP; and (iii) a large reserve pool (RP), making up 80%–90% of SVs, thought to serve as a storage compartment located away from the active zone. The dynamic interchange among these different pools is orchestrated by synapsins, a family of phosphoproteins that regulate the tethering and release of SVs from the actin cytoskeleton (Longhena et al., 2021). On the postsynaptic zone, the clustering of receptors and channels is facilitated by post synaptic density proteins, including PSD95, along with several enzymes involved in actin polymerization. These elements contribute to changes in spine morphology and modulate the postsynaptic response to stimuli (Hlushchenko et al., 2016).

Despite the limited space within synaptic structures, their fine regulation is ensured by both local protein synthesis and axonal transport (anterograde and retrograde) of various synaptic precursors, since the primary biosynthetic machinery resides in the soma and dendritic compartments, often located far from the synapse (Rizalar et al., 2021). In addition to the modulation of the transport of proteins and RNA necessary for these zones, regulation is based on the control of protein activity, protein-protein interaction and balance between protein stability and degradation, mainly achieved by post-translational modifications (PTMs). Among these, the Small ubiquitin-like modifier (SUMO) conjugation (SUMOylation) is emerging as dynamic PTM involved in the regulation of synaptic proteins thus contributing to the modulation of synapse structure and function as well as to synaptic transmission and plasticity. In this context, we will explore the physiological impact of SUMOylation and how its dysregulation plays a central role in Alzheimer’s disease, serving as a representative example of neurodegenerative disorders.

### SUMOylation enzymatic pathway

Proteins undergoing SUMOylation are modified by the covalent attachment of the SUMO peptides, which are small proteins ranging from 10 to 20 kDa. To date five paralogs have been identified: SUMO1, SUMO2, SUMO3, SUMO4, and SUMO5 with only SUMO1-3 being expressed in the brain. SUMO2 and 3 have 95% of homology and are often collectively referred to as SUMO2/3. In contrast, they share only about 45% homology with SUMO1, indicating functional divergence. While SUMO 4 and 5 are less characterized and their implication in pathophysiological processes is still under investigation, SUMO1-3 are well-established as key regulators of neuronal function (Matsuzaki et al., 2015; Datwyler et al., 2011).

Indeed, regulation of SUMOylation balance (SUMO1 versus SUMO2/3) or levels (SUMOylation versus deSUMOylation) is a crucial determinant of neuronal development and differentiation (Pronot et al., 2021; Bernstock et al., 2019; Du et al., 2020) plasticity (Du et al., 2020) and synaptic transmission (Martin et al., 2007). This balance is maintained through the interplay between the SUMOylation cascade and the deSUMOylating enzymes, which include three main families of serine proteases (SENP, sentrin-specific protease; deSUMOylating isopeptidase; USPL1, ubiquitin-specific peptidase-like protein 1) that remove SUMO peptides from target proteins.

**FIGURE 1:**
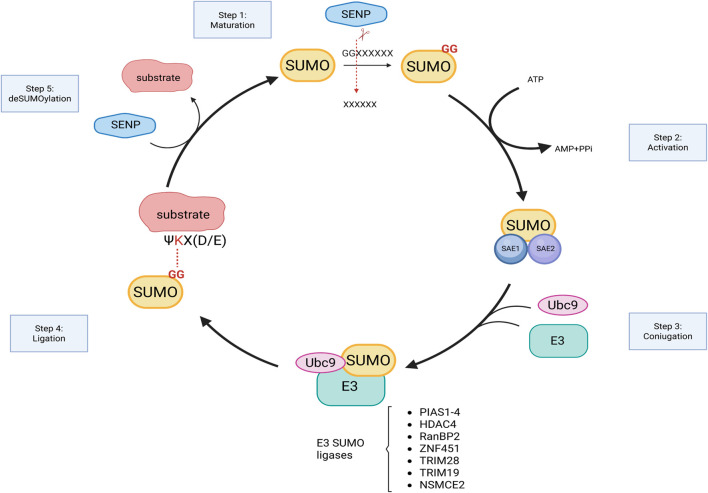
Graphical representation of SUMOylation enzymatic cascade. SUMO, synthesized as precursor, is cleaved by SENPs to expose diglycine (GG) motif of SUMO at the C-terminus. Mature SUMO, bound to E1 activating enzymes, is then ready to be conjugated to the target by the sequential action of Ubc9 and E3 ligase enzymes (examples are listed). SUMO attachment is reversible and is catalyzed by SENPs. Figure created with Biorender.

During the SUMOylation enzymatic cascade (Figure 1), SUMO proteins are covalently attached to the target substrates via a sequential reaction that involves: maturation of the SUMO protein that consists in the cleavage of the C-terminus, operated by SUMO-specific proteases, that expose the Gly-Gly motif (SUMO-GG), necessary for SUMO ligation (Johnson et al., 1997); activation of the mature SUMO achieved through the binding of the SUMO-GG to the E1 enzyme (SAE1/SAE2) in an ATP-dependent manner; transfer of the activated complex to the active cysteine site of Ubc9 that facilitates SUMO attachment to the target with the assistance of the E3 ligase enzyme. While Ubc9 is the only E2 enzyme identified so far, a growing number of E3 ligases have been characterized. They can be grouped on the basis of structure and activity domains:

1. The SP-RING domain family that comprises PIAS proteins (PIAS1-4) whose ligation activity is dependent on the presence of the E2 enzyme (Jackson, 2001); Beyond SUMOylation, PIAS proteins are also involved in other cellular processes such as transcriptional regulation, DNA repair, and nuclear-cytoplasmic transport (Rytinki et al., 2009; Galanty et al., 2009; Sachdev et al., 2001; Kagey et al., 2003; Pichler et al., 2002).
2. The TRIM (Tripartite Motif) superfamily includes proteins characterized by the presence of a tripartite motif responsible for E3 ligase activity. These proteins also present additional domains such as B-box and coiled-coil domains important for self-aggregation and additional functions in immunity (Ozato et al., 2008).
3. The SIM-Containing SUMO E3 Ligases, lack both RING and TRIM motifs and mediate SUMOylation through a SUMO-interacting motif (SIM). SIM is a short sequence of hydrophobic residues flanked by serine or acidic residues typically located at either the N- or C-terminus. This motif allows non-covalent interactions between SUMOylated proteins and their binding partners. Nucleoporin RanBP2 (Pichler et al., 2002), a component of the nuclear pore, and Pc2, a component of polycomb group (PcG), both belong to this category (Merrill et al., 2010; Reverter and Lima, 2005). Interestingly, within the nuclear pore complex, the nucleoporin 153 has been reported to anchor the deSUMOylating enzymes SENP1 and 2 (Chow et al., 2012) thereby contributing to balance the RanBP2-dependent SUMOylation of nuclear transcription and epigenetic factors as well as proteins destined for export to the cytoplasm.
4. Non canonical E3 ligase activity has also been identified in several proteins that lack the RING or TRIM domains and do not possess a SIM module. In these cases, the specific region for SUMOylation activity has yet to be fully characterized. Among these proteins, the histone deacetylase 4 (HDAC4) and 7 (HDAC7) have been identified as SUMO E3 ligases. HDAC7 has been shown to promote the SUMOylation of Promyelocytic leukemia protein (PML) thereby facilitating the formation of PML nuclear bodies (Gao et al., 2008). HDAC4, on the other hand, has a broader range of identified SUMOylation targets. For instance, it inhibits NF-kb interacting with Ubc9 and by increasing the SUMOylation of the inhibitory subunit IkBa (Yang et al., 2020); suppresses androgen receptor by its SUMOylation (Yang et al., 2011); stabilizes SIRT1 and delays senescence (Han et al., 2016); inhibits MEF activation through SUMOylation (Zhao et al., 2005). More recently, HDAC4 has been identified as E3 ligase regulating SUMO2/3 levels in the synaptic compartment (Colussi et al., 2023).

Importantly, for both HDAC7 and HDAC4, the SUMO ligase activity is independent from their deacetylase activity, although the precise domains responsible for SUMOylation remain to be defined.

### Pharmacological modulation of SUMOylation

Given the central role of SUMOylation in physiology and its deregulation in several neurodegenerative diseases, numerous inhibitors and activators have been developed with the aim of promoting neuroprotection. In pathological contexts, depending on the substrate and the type of SUMO added (SUMO1 versus SUMO2/3), SUMO conjugation can be either beneficial or detrimental. A wide range of small molecules with inhibitory activity against the E1 and E2 SUMOylation enzymes have been identified; however, no inhibitors targeting SUMO-E3 ligases have been discovered to date. The compounds identified include natural products, peptidomimetics, and synthetic derivatives obtained through virtual screening (for an extensive review see ref. (Brackett and Blagg, 2021).

For example, among the inhibitors of the E1 enzyme are both natural products (e.g., ginkgolic acid, anacardic acid, tannic acid) and synthetic compounds (e.g., phenyl urea, pyrazole urea, and thiazole urea) that block the formation of the E1–SUMO complex (Fukuda et al., 2009; Kumar et al., 2013). Other molecules, instead, induce conformational changes that prevent the binding of E1 with ATP and SUMO (Lv et al., 2018).

The second step of the SUMOylation cascade is carried out by Ubc9, the only known E2 enzyme. Natural inhibitors of this enzyme include spectomycin B1, chaetochromin A, and viomellein (Hirohama et al., 2013). The flavone 2-D08 also targets Ubc9 by inhibiting the transfer of SUMO from Ubc9 to the substrate (Kim et al., 2013). Synthetic derivatives of the SUMO consensus region have also been used to develop peptides capable of competing with SUMO1 for Ubc9 binding.

So far, relatively few studies have focused on the development of SUMO activators compared to SUMO inhibitors. One of the first molecules identified as a SUMO activator is a negative regulator of miR-182 and miR-183, whose inhibition leads to a global increase in both SUMO1 and SUMO2/3 levels (Bernstock et al., 2016). This molecule also demonstrated neuroprotective effects *in vitro*.

N106 is another small molecule identified through screening, which selectively enhances SUMO1-ylation of the cardiac sarcoplasmic reticulum calcium ATPase (SERCA2a). Treatment with N106 was shown to restore cardiac function in a mouse model of heart failure (Kho et al., 2015). Notably, the authors did not observe a significant increase in overall SUMO1-ylation following N106 treatment, with only limited additional targets affected in cardiomyocytes. Nevertheless, since the mechanism of action of this molecule involves activation of the E1 enzyme, it is possible that other targets may be identified in different organs, such as the brain, which could be exploited to modulate neuronal function.

In a subsequent study, using a high-throughput homogeneous time-resolved fluorescence (HTRF) assay, several SUMO activators were identified, belonging to three chemical classes: quinolines, benzothiazoles, and aminothiazoles (Krajnak and Dahl, 2018). The degree of SUMO activation varied among these classes and their subtypes (43% for quinolines, 38% for benzothiazoles, and 53% for aminothiazoles). Nevertheless, all optimized compounds within each category demonstrated *in vitro* neuroprotection against ER stress-induced cell death, suggesting potential therapeutic applications for neurodegenerative diseases. Another way to increase SUMOylation is by blocking SENPs, thereby preventing the removal of SUMO from proteins (Albrow et al., 2011; Wang et al., 2020). Recently, this class of small molecules has also demonstrated neuroprotective effects in brain ischemia, where drug-induced increases in both SUMO1- and SUMO2/3-conjugated proteins improved recovery following stroke. Further studies will be necessary to evaluate the potential use of these compounds in the context of neurodegeneration as well (Yang et al., 2016).

Important steps toward the *in vivo* use of all these promising compounds, whether activators or inhibitors, include determining their bioavailability, tissue solubility, ability to cross the blood-brain barrier, and, in some cases, identifying the specific SUMO enzyme (E1, E2, or E3) they target. Moreover, deregulation of the SUMO machinery in disease may result in either a global increase in SUMO1 or SUMO2/3 conjugation, or selective SUMOylation/deSUMOylation of specific targets. Therefore, it is crucial to assess how these small molecules affect both global and target-specific SUMOylation, as well as their potential off-target effects.

### Effects of SUMOylation on protein function

**FIGURE 2:**
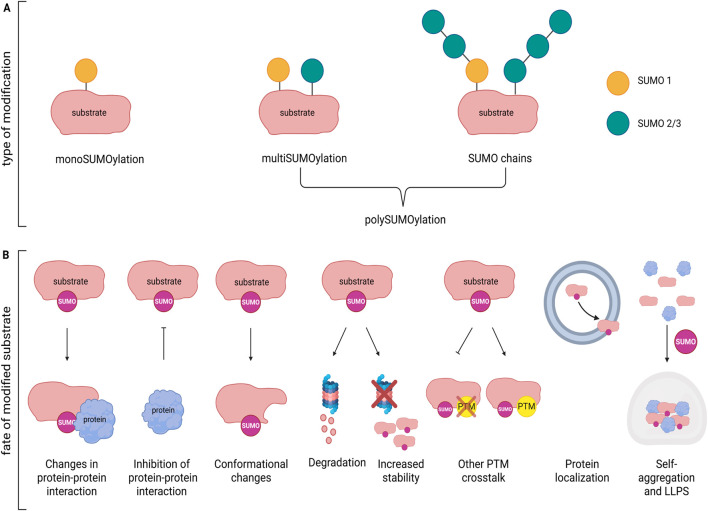
Effects of SUMOylation on protein function. **(A)** Example of mono- and poly-SUMOylation by SUMO1, SUMO2/3 or both on a substrate. **(B)** Substrate modification by SUMO may modify protein function in multiple ways: inducing changes in protein-protein interaction; inhibiting protein binding; determining conformational changes; affecting stability/degradation; favoring or inhibiting other PTMs; modulating protein localization; modifying self-aggregation capacity and formation of LLPS. Figure created with Biorender.

Some proteins are modified exclusively by SUMO1 or SUMO2/3, while others can undergo both modifications via the same catalytic pathway. Typically, SUMO1 is added as single unit to the substrate (mono-SUMOylation), whereas SUMO2/3 units are attached as chains (poly-SUMOylation). However, in certain cases SUMO1 itself can be further be modified by the addition of SUMO2/3 chains (Vertegaal et al., 2006) (Figure 2A). Modification occurs preferentially at a consensus site defined as (Ψ-K-X-[(D/E] (Tatham et al., 2001), where the lysine to be SUMOylated (K) is preceded by a large hydrophobic residue Ψ, while X can be any residue followed by aspartate or glutamate (D/E) (Rodriguez et al., 2001). Nevertheless, it has been observed that SUMOylation may occur in other non-canonical consensus (Hietakangas et al., 2006). Furthermore, PTMs in surrounding sequence or targeting the same lysine may enhance or compete with SUMOylation. For instance, acetylation and SUMOylation can be mutually exclusive when they target the same lysine, whereas phosphorylation of a nearby serine residue can enhance SUMOylation (Sapetschnig et al., 2002).

Addition of SUMO to the target protein changes its inter- and/or intramolecular interactions, often inducing conformational changes that may mask or expose nuclear localization/export (NLS/NES) sequences, or change affinity for specific transporters/adaptors thereby influencing transport between the nucleus and cytoplasm or direction through particular cellular compartments (e.g., dendritic spines). For example, SUMOylation of the serine/threonine kinase GSK3β, a key player in neuronal morphology, synapse formation and neuroinflammation, leads to its nuclear import (Eun Jeoung et al., 2008). Local translation at synapses depends on the availability of specific mRNAs that are packed and transported along the axons by the microtubule network. The RNA-binding protein La recognizes specific tagged-mRNA and transport them in anterograde or retrograde direction depending on its SUMOylation status (van Niekerk et al., 2007). SUMOylated La is dedicated to retrograde transport towards the nucleus, while deSUMOylated La allows anterograde transport by binding to kinesin. Changes in functional modulation (Figure 2B) are also common effects after SUMOylation. If SUMO modification of the protein/enzyme occurs close to the active site it may inhibit the activity by blocking substrate access or may enhance the activation by stabilizing a catalytically favorable conformation. For example, SUMO1 conjugation at lysine 430 within the catalytic domain of protein phosphatase 5 (PP5), activated during stress-induced signaling, regulates its enzymatic activity and substrate release (Sager et al., 2024). SUMOylation can also enhance or inhibit interactions with binding partners. SUMO-dependent modification of the interaction with cofactors or regulatory proteins, as well as prevention of other activatory/inhibitory PTMs (e.g., ubiquitination, phosphorylation) may likewise modify protein activity. Moreover, many proteins can bind SUMO non-covalently through SUMO-interacting motif (SIM), a sequence containing a hydrophobic core flanked by acidic or phosphorylatable serine residues. The SUMO/SIM interaction acts as a platform for other partners thus expanding the protein network fundamental for the assembly of large molecular transcriptional complexes involved in epigenetic regulation. HDAC4 dependent SUMO1-ylation of MeCP2, for example, activates the HDAC4/MeCP2 repressive complex during neuroinflammation (Li Puma et al., 2023), while SUMO-1 modified RAD51 promotes Sp1 transcriptional activity (Wu et al., 2025).

In general, SUMOylation increases protein stability by preventing ubiquitination at the same lysine residue, thereby protecting the protein from proteasomal degradation (Wei et al., 2025).

In other contexts, stress-driven SUMOylation, may dramatically alter protein solubility and promote self-aggregation, especially in proteins with intrinsically disordered domains, that are more prone to condensate and to be incorporated into stress granules, dynamic RNA-containing condensates (Verde et al., 2025). For example, preventing SUMO2/3-ylation of TDP43, a protein involved in amyotrophic lateral sclerosis and frontotemporal dementia, prevents its cytosolic aggregation and formation of stress granules. However, the formation of molecular condensate is not always deleterious. In fact, it can serve as a mechanism to compartmentalize groups of proteins or enzymatic pathways within small, membrane-less regions via liquid-liquid phase separation (LLPS) (Guzikowski and Kavalali, 2024), a process that may be particularly crucial at the level of the synaptic nanodomains.

Overall, these findings underscore the critical role of SUMOylation in a wide range of biological processes, including those essential for neuronal function. Dysregulation of SUMOylation may therefore contribute to the pathogenesis of various neurological disorders.

## SUMOylation dependent regulation of synapse function

### Functional aspects

SUMOylation is highly abundant in the nucleus and indeed many SUMO substrates, including transcription factors and co-regulators, were first identified in this compartment (Vertegaal, 2022).

Also, the SUMOylation machinery including E1-E3 enzymes and SENP proteases are present in the nucleus and are localized in the nuclear matrix where they play roles in processes such as chromosome segregation (Chen et al., 2024). Additionally, components of the SUMOylation system are associated with the nuclear pore complex (Chow et al., 2012; Ptak et al., 2025), where protein import and export are tightly coupled with SUMOylation, significantly influencing protein localization and function. For example, nuclear localization of the Downstream regulatory element antagonist modulator (DREAM), a multifunctional Ca^2+^-sensitive protein, occurs upon SUMO1-ylation and enables its repressive activity on several targets including sodium/calcium exchanger isoform 3 (NCX3), brain-derived neurotrophic factor (BDNF), prodynorphin, and c-fos (Palczewska et al., 2011). Furthermore, SUMOylation of TDP-43, a nuclear RNA-binding protein that forms toxic aggregates in amyotrophic lateral sclerosis and frontotemporal dementia patients, promotes its nuclear export and aggregation in the cytoplasm within stress granules (Maraschi et al., 2021).

Although less abundant outside the nucleus, SUMOylation plays a key role in synaptic function and development. SUMOylation enzymes and SENPs have been shown to localize not only in the cytoplasm but also specifically at synapses. For example, SENP5 immunoreactivity has been observed both in the postsynaptic spines and at the presynaptic zone (Akiyama et al., 2018), while by means of super-resolution microscopy the colocalization of SUMO1, SUMO2/3, and Ubc9 with pre- and post-synaptic markers was demonstrated (Colnaghi et al., 2019). The dynamic role of SUMOylation at synapse is also indicated by estrogen-dependent redistribution of Ubc9 and its interaction with PSD95 and synaptophysin in the Brain of APP/PS1 mice, a model of AD, and cortical neurons as revealed by immunofluorescence and co-immunoprecipitation (Lai et al., 2017).

HDAC4, an enzyme with low deacetylase activity, has recently been described as a new E3 ligase enzyme that localizes at synapses. It modulates the enrichment of PSD95 at the post-synaptic membrane by increasing its SUMO2/3ylation conjugation thus promoting synaptic transmission (Colussi et al., 2023). Moreover, activity-dependent Ubc9 localization to dendritic spines has been observed downstream metabotropic glutamate 5 receptors (mGlu5R) activation, process that leads to increased synaptic SUMOylation (Loriol et al., 2014).

In the same context, activity-induced Akt1 SUMO1-ylation has been reported as necessary for activation of the ERK1/2-BDNF/Arc signalling cascade, which supports LTP and long-lasting excitatory synaptic responses (Meng et al., 2021). Spatiotemporal regulation of synapse SUMOylome is crucial not only during normal synaptic activity but also throughout development and synaptogenesis. Indeed, proteomic studies on the developing brain have shown that the SUMO2/3-ylome of the synaptic compartment is enriched in neurotransmitter receptors, adhesion molecules, scaffolding proteins, molecules involved in vesicular trafficking and cytoskeleton rearrangement, all necessary for synapse maturation (Pronot et al., 2021).

To date, numerous pre- and post-synaptic proteins, including ion channels, receptors and transducers have been identified as either SUMO substrate or SUMO-interacting protein widening the complexity of the SUMOylome network that regulates these highly specialized compartments.

On the presynaptic side, several proteins involved in the synaptic vesicle cycle are modulated by SUMOylation. Synapsin I, one of the main regulators of synaptic vesicle pool availability, is subject of SUMO1 modification that increases its binding to SVs and promotes their reclustering after stimulation thereby contributing to the maintenance of the reserve pool (Tang et al., 2015). Of note, synapsin I is also target of many kinases and can be phosphorylated at various sites, critical for its interaction with actin and SVs, or for their release in the fine-tuning of synapsin I function (Longhena et al., 2021).

Efficient and controlled exocytosis of SVs is also regulated by the SUMO1 conjugation of the Rab3-interacting molecule 1α (RIM1α). This modification promotes the clustering of Ca_V_2.1 calcium channels, so enhancing Ca^2+^ influx required for vesicle release (Girach et al., 2013). SV fusion with the plasma membrane is regulated by the interaction of SNARE proteins, located on both the SV and the presynaptic membrane at the active zone. SUMO1-ylation of Syntaxin1, a core component of the SNARE complex, increases its interaction with other SNARE proteins promotes endocytosis, suggesting an additional role for SUMO-modified Syntaxin1 in regulating the rate of neurotransmitter vesicle recycling (Craig et al., 2015). However, SUMO1-ylation can also have detrimental effects on synaptic function. In a study using mice with a neuronal restricted SUMO1 expression, the authors found an altered basal synaptic transmission and impaired presynaptic function that were associated with increased SUMO1-ylation of synaptotagmin-1 (Matsuzaki et al., 2015). Although the precise role of SUMOylation on synaptotagmin-1 was not fully elucidated, these findings suggest that that elevated SUMO1-ylation may have a role in altered neuronal function and neurodegenerative disease mechanisms.

According to the most recent models of presynaptic compartmentalization, synaptic vesicles (SVs) and the proteins involved in the entire SV cycle, including exocytosis and endocytosis, are organized into distinct or overlapping membrane-less assemblies based on liquid-liquid phase separation that is driven by the presence of disordered regions and SUMOylation (Zhang et al., 2024). This suggests that presynaptic protein SUMOylation may be essential for the formation of dynamic sub-synaptic protein clusters, enabling the fine-tuning of the SV cycle in response to neuronal activity and external stimuli.

The postsynaptic zone is another small yet highly specialized compartment, responsible for receiving neurotransmitter and converting the chemical signal into electrical one. The more specialized region is the post synaptic density (PSD), a protein-dense structure that gathers receptors, signaling molecules and scaffold proteins, many of which have been found to be SUMO2/3ylated by a proteomic approach in rat brains at the post-natal day 14 (Pronot et al., 2021). For example, β-catenin, PSD95, SynGAP, SAPAP3, Homer1, CaMKII, Neuroligin and N-Cadherin have all been found to undergo SUMOylation, although the functional consequences of these modifications remain largely unexplored. More recently, in adult mice, SUMO2/3-ylation of PSD95, the core component of PSD, has been observed. This modification is regulated by a cytoplasmic pool of HDAC4, which acts as an E3 ligase. SUMOylation of PSD95 was shown to promote its anchoring to the postsynaptic membrane, suggesting a role in stabilizing synaptic architecture (Colussi et al., 2023).

Membrane remodeling at the post synaptic density zone is fundamental to structural changes in dendritic spines and include receptor membrane trafficking, cytoskeletal rearrangement and activation of several signaling pathways that coordinate these processes (Hotulainen and Hoogenraad, 2010; Nishiyama and Yasuda, 2015; Spence and Soderling, 2015). Many of the proteins involved in actin remodeling and spine morphology regulation are modulated by SUMOylation. For example, CaMKII (calcium/calmodulin-dependent protein kinase II) activated by increased Ca^2+^ influx upon synaptic stimulation, phosphorylates several targets including the activity-regulated cytoskeleton-associated protein (Arc) that plays a key role in driving actin remodeling. Upon stimulation, Arc mRNA is rapidly translated locally at synapse and undergoes dynamic mono-SUMO1-ylation that is necessary for its interaction with the actin binding protein debrin (DaSilva et al., 2016; Lyford et al., 1995; Nair et al., 2017). Furthermore, Arc interacts with cofilin, one of the main proteins that regulates actin polymerization (Messaoudi et al., 2007). On the contrary, deSUMOylated Arc is associated with decreased F-actin stabilization and spine size (Newpher et al., 2018).

Kainate receptors (KARs) represent another example of SUMO-regulated receptors. KARs are glutamate receptors that, depending on the neuronal type, present a distribution at pre- and post-synaptic sites and in extrasynaptic regions. Their signalling may occur either as ionotropic channels or metabotropic receptors based on G protein-coupled signals. Due to this broad distribution and functional versatility, KAR are modulators of neuronal excitability and synaptic transmission. These receptors are composed of tetrameric assemblies of different subunits (GluK1-5), among which the Gluk2 subunit is regulated by multiple PTMs including SUMO1-ylation (Martin et al., 2007). Indeed, Gluk2 SUMO1-ylation is necessary for receptor internalization upon agonist binding. Interestingly, interplay among SUMOylation, phosphorylation and palmitoylation coordinates KAR surface expression and activity-dependent endocytosis. Under basal (unstimulated) conditions, palmitoylation of GluK2 inhibits both phosphorylation and SUMOylation, thereby stabilizing the receptor at the plasma membrane. However, upon kainate stimulation, Gluk2 is depalmitoylated and undergoes phosphorylation and SUMOylation that in turn stimulates internalization (Yucel et al., 2023; Chamberlain et al., 2012; Konopacki et al., 2011).

K2P family channels form K^+^ selective pores that regulate the background flux of potassium ions that are key determinants of the resting membrane potential. Two homomeric or heteromeric subunits (there are 145 genes so far identified) assembly to form the pore (Plant et al., 2012; Lopes et al., 2001). Although it has been object of debate, it has been demonstrated that a basal SUMO1-ylation of the K2P1 isoform maintains the channel in an inactive state (Rajan et al., 2005). Accordingly, a co-localization of K2P1, SAE1, and Ubc9 was observed at the plasma membrane, and conjugation of SUMO1 to the ε-amino group of Lys274 was also confirmed by a proteomic approach (Plant et al., 2010). Furthermore, when SUMOylated-K2P1 is associated with K2P3 or K2P9 subunits at the surface of cerebellar granule neurons their response to stimuli is suppressed indicating a more wide effect of SUMOylation on these channels (Plant et al., 2012).

The regulatory role of SUMOylation on potassium channel was also demonstrated by means of SENP2-deficient mice that exhibit seizures and sudden death (Qi et al., 2014). In this model deficiency of SENP2 led to hyper-SUMOylation of Kv1.1 and Kv7.2/Kv7.3 channels. Changes in SUMO levels however affected only Kv7.2/Kv7.3 channels causing a reduction in depolarizing M-current responsible for neuronal hyperexcitability.

Several sodium channel isoforms (Na_V_1.1, Na_V_1.2, and Na_V_1.6) are present in mature neurons with a specific spatiotemporal distribution that ensures proper onset and propagation of action potential (AP). In particular, Na_V_1.2 channels, located in the proximal part of the axon initial segment, where AP originates, is responsible for backpropagation of AP to the soma/dendrites important for synaptic strength and the coordination of synaptic inputs (Markram et al., 1997).

SUMO1-ylation of Na_V_1.2 has recently found as critical modification able to increase action potential backpropagation from the AIS providing a new layer of complexity in the modulation of synaptic integration and plasticity (Kotler et al., 2023). Additionally, SUMO1-ylation of Na_V_1.2 channels occurs in response to brain hypoxia and allows a neuroprotective response to this stress increasing sodium current (Plant et al., 2016).

Overall, these works outline the role of sodium channel SUMOylation as important means to modulate neuronal response to different stimuli.

### Structural aspects

The type and the magnitude of synaptic transmission rely on the signalling generated between the pre- and post-synaptic compartments which is strongly influenced by the shape, size, and organization of these specialized sites. For example, several structural proteins and their regulators contribute to the maturation, stabilization, or retraction of dendritic spines. These formations are enriched in actin filaments and microtubules which are continuously modulated in terms of polymerization, organization, stability, and turnover ensuring rapid response to stimuli. Activity-dependent changes in actin polymerization enable spines to change shape, enlarge or become stabilized thus supporting learning processes and memory storage (Kasai et al., 2003).

The calcium/calmodulin-dependent serine protein kinase (CASK) belongs to the family of the membrane-associated guanylate kinase (MAGUK) proteins. CASK has been identified as a key element inducing actin nucleation, assembly and spine formation through its interaction with the adhesion protein 4.1 (Biederer and Sudhof, 2001). However, it has been shown that SUMO1-ylation of CASK at lys 679, alters the binding site for protein 4.1 reducing the formation of the complex actin/protein4.1/CASK that leads to a decrease in the number and size of spines (Chao et al., 2008).

Other studies indicate that the control of actin at the level of spines may be obtained by regulating its transcription. Indeed, local translation within these small compartments is fundamental for achieving precise spatial and temporal regulation of structural and signaling proteins. Specifically, actin interacts in neurons with the prion-like cytoplasmic polyadenylation element-binding protein 3 (CPEB3) (Stephan et al., 2015), that is an RNA binding protein identified as an important modulator of long-term synaptic plasticity in the hippocampus (Fioriti et al., 2015).

Under basal conditions CPEB3 is SUMO2/3-ylated, remains soluble and acts as repressor of mRNA translation (Drisaldi et al., 2015). Upon neuronal stimulation, CPEB3 is deSUMOylated and undergoes aggregation which promotes the translation of its target mRNAs, including actin, ultimately influencing spine structure and synaptic plasticity (Gu et al., 2020).

Dynamic interaction of actin with the microtubule cytoskeleton is another key element in maintaining spine morphology and maturation of synapses (Dent and Gertler, 2003). In this context, Spastin is a protease that fragments microtubules in an ATP dependent manner when SUMO1-ylated at K427. Conversely, its deSUMOylation leads to microtubule stability (Ji et al., 2020). Since only dynamic microtubules can enter the dendritic spines and regulate F-actin levels it is evident that SUMOylation balance of Spastin is crucial for maintaining microtubule polymerization dynamics.

Altogether, the coordinated regulation of both structural and functional elements is fundamental for the remodeling and maturation of dendritic spines, processes that are essential for synaptic plasticity and, ultimately, cognitive function.

### Deregulation of SUMOylation in Alzheimer’s disease

Thanks to its dynamic nature, SUMOylation enables the rapid and reversible modification of protein function in response to signals occurring well before changes in gene expression. This mechanism underlies several neuronal processes including the regulation of synaptic transmission and plasticity (Loriol et al., 2012; Watanabe et al., 2008). Indeed, increase in SUMO1 conjugation and in Ubc9 levels characterize the early developing brain while activity-dependent higher SUMOylation and redistribution of the SUMO machinery occur both at the level of pre- and post-synaptic compartments facilitating neuronal signal transmission (Loriol et al., 2013). In addition, SUMOylation-dependent changes are necessary for long-term synaptic plasticity (LTP). Indeed, following stimulation, SUMO2/3 levels transiently peak, but acute inhibition of SUMOylation impairs both LTP and hippocampal-dependent learning (Lee et al., 2014).

Because of its key role in modulating protein function and neuronal signaling, disruption of SUMOylation homeostasis, whether through excessive conjugation or impaired deSUMOylation, is linked to several neurological disorders including Alzheimer’s disease (AD). AD is the most common neurodegenerative disorder in the elderly characterized by increased deposition of amyloid beta (Aβ), which accumulates in plaques, and by increased levels of phosphorylated tau leading to the formation of neurofibrillary tangles. Synaptic failure is an early event in the pathogenesis of the disease that represents the best correlate of disease progression and is considered a key determinant of its severity (Selkoe, 2002; Reddy et al., 2005).

Dysregulation of SUMOylation has been observed in Alzheimer’s disease (AD) patients and demonstrated across multiple animal models of pathology. Both human subjects and AD models show an altered global SUMOylation profile, with generally elevated levels of SUMO1 and reduced levels of SUMO2/3 (Li et al., 2003; Lee et al., 2014). Elevated SUMO1 levels have also been detected in plasma samples from AD patients, suggesting a potential role as a biomarker (Cho et al., 2015). Importantly, deregulation of SUMO balance was linked to impaired LTP and hippocampal-dependent learning and memory (Lee et al., 2014). Accordingly, transgenic mice with SUMO1 restricted overexpression in the brain exhibited altered basal synaptic transmission and impaired presynaptic function (Matsuzaki et al., 2015). Of note, altered SUMOylation has been observed both in post-mortem AD brain and in presymptomatic patients (Mandel and Agarwal, 2022) thus suggesting that changes in SUMOylation is more a chronic status rather than an acute episode.

Focusing on the SUMOylation of specific targets, several reports have shown its impact on the two pathological AD hallmarks, Aβ and tau. Amyloid beta is generated through the amyloidogenic processing pathway that involves that sequential cleavage of the amyloid precursor protein (APP) by β-site APP cleaving enzyme (BACE), followed by γ-secretase (Wolfe and Haass, 2001). Different effects on Aβ deposition and aggregation have been reported depending on the type of SUMO modification and protein targets involved. Increasing the SUMO3 expression in an *in vitro* system, for example, induced a reduction of Aβ production (Li et al., 2003) and APP SUMO1 conjugation at lysines 587 and 595, close to the site of BACE cleavage, reduced Aβ aggregation (Zhang and Sarge, 2008). Conversely, SUMO1-ylation of BACE1 at lysine 501 enhanced the enzyme’s protease activity and stability, resulting in elevated Aβ production (Bao et al., 2018). Tau is a microtubule associated protein that undergoes aggregation in several neurodegenerative disorders including AD. This protein is target of many PTMs, in particular phosphorylations, that change its solubility and promote aggregation. Recent advances led to the discovery that interplay among different PTMs can either exacerbate or mitigate tau aggregation and its associated pathology. Specifically, SUMO1-ylation of tau increased the level of its phosphorylation while reducing its solubility (Wada et al., 2024). Conversely, SUMO2 modification of tau enhances its solubility and reduces tau-dependent pathological effects (Zhang et al., 2023).

Additional evidence of the contrasting effects resulting from the imbalance of SUMO1- and SUMO2/3-conjugated proteins is provided from the altered localization and function of the E3 ligase enzyme HDAC4. In AD, where HDAC4 is mislocalized from synapses and accumulates in the nucleus, there is a reduction in the SUMO2/3-ylation of PSD95, one of its targets, that is associated with the loss of membrane localization of several proteins important for synaptic transmission such as CaMKII, GluA1, N-cadherin (Colussi et al., 2023). HDAC4 nuclear accumulation also characterizes an AD-like model induced by neuroinflammation. In this model, HDAC4 mediates the SUMO1 conjugation of the transcriptional repressor MeCP2, resulting in the formation of a multimeric complex containing HDAC4 and MeCP2 that suppresses the expression of synaptic genes (Li Puma et al., 2023).

Based on these data several research groups developed strategies to modulate SUMOylation as a means of restoring synaptic function. Downregulation of SUMO1-ylation in the synaptosomal compartment successfully normalized impaired glutamate release further highlighting the detrimental effects of elevated SUMO1 levels (Marcelli et al., 2017). Conversely, AD mice (APP) either overexpressing SUMO2 or treated with a brain-penetrant recombinant SUMO2 showed a recovery in cognitive and synaptic impairment along with reduced amyloid pathology suggesting that a pharmacological approach may be a promising therapeutic tool (Fioriti et al., 2025). Similarly, viral induced expression of SUMO2 rescued neuronal toxicity in mice overexpressing the mutant form of tau (Orsini et al., 2024).

## Conclusion

Protein modification through SUMOylation plays a crucial role in regulating their function inducing conformational changes or masking/exposing sites critical for protein-protein interaction or influencing other post-translational modifications (PTMs). These actions collectively affect protein stability, degradation, solubility, and activity. Synaptic function is regulated by SUMOylation both at the nuclear level and directly at synapses, where an increasing number of proteins have been identified as SUMO targets. Consequently, alteration in SUMO machinery, frequently observed in many neurodegenerative diseases, may have a central role in disease pathogenesis. Specifically in AD, reducing SUMO1-ylation or increasing SUMO2-ylation demonstrated a beneficial effect on synaptic function suggesting that targeting SUMOylation could represent a promising therapeutic approach for restoring synaptic health in AD.
